# Acute multiple lesions resulting from segmental arterial mediolysis: An autopsy case report of subarachnoid hemorrhage accompanied by great omentum bleeding

**DOI:** 10.1097/MD.0000000000043533

**Published:** 2025-08-01

**Authors:** Miu Shimamoto, Kenji Ninomiya, Akira Yogi, Kazumichi Kakazu, Natsuki Ikematsu, Maki Fukasawa, Mio Takayama

**Affiliations:** a Department of Legal Medicine, Graduate School of Medicine, University of the Ryukyus, Ginowan, Okinawa, Japan; b Department of Radiology, University of the Ryukyus Hospital, Okinawa, Japan; c Criminal Investigation Laboratory, Okinawa Pref. Police H.Q., Okinawa, Japan.

**Keywords:** acute abdomen, aneurysm, autopsy, segmental arterial mediolysis, subarachnoid hemorrhage

## Abstract

**Rationale:**

This report describes a rare case of segmental arterial mediolysis (SAM) in a previously healthy man with no known underlying medical conditions. The patient had presented with simultaneous subarachnoid hemorrhage (SAH) and hemorrhage of great omentum, an atypical combination that highlights the diagnostic challenges and severity of SAM. The report outlines the disease’s clinical course, autopsy findings, and computed tomography angiography results.

**Patient concerns:**

A man in his 40s with no history of chronic illness, presented with symptoms of acute abdomen and intracranial hypertension. Despite receiving medical attention, he died shortly after admission.

**Diagnoses:**

The patient was diagnosed with suspected enteritis before his death.

**Interventions:**

An autopsy was performed to examine the cause of death.

**Outcomes:**

Postmortem examination revealed an acute SAH secondary to a ruptured vertebral artery aneurysm and acute hemorrhage of the greater omentum. The splenic artery was highly tortuous and dilated. Histopathological examination confirmed involvement of the SAH, greater omentum hemorrhage, and splenic artery lesions, suggesting a common underlying vascular pathology for these simultaneous hemorrhagic events.

**Lessons:**

The case underscores the diagnostic challenges associated with SAM due to its varied clinical presentations and unpredictable symptom progression. It also emphasizes the importance of early detection and treatment of SAM for the prevention of further complications.

## 1. Introduction

Segmental arterial mediolysis (SAM), described by Slavin et al in 1976,^[1]^ is a systemic, non-atherosclerotic, and noninflammatory arterial degenerative disease. SAM can cause abdominal aneurysms once the arterial tunica media fuses and degenerates, leading to a fragile vessel wall and, eventually, a fatal hemorrhage. SAM is associated with a 22% to 26% mortality rate,^[2,3]^ but the cause of the disease remains unknown. SAM is more common in men,^[2,4]^ with an incidence of approximately 1 in 100,000.^[4]^ Recent advancements in imaging techniques have led to an increase in reported cases as they facilitate more accurate diagnosis.

SAM can affect arteries throughout the body. Intra-abdominal hemorrhage accompanied by vertebral artery dissection and death resulting from subarachnoid hemorrhage (SAH) caused by vertebral artery rupture have been reported following autopsies.^[5,6]^ Cases of intra-abdominal or pelvic hemorrhage during the course of SAH have also been documented.^[7–9]^ Because of mixed old and new vascular lesions in various vessels, diagnosis can be challenging owing to diverse presentations and the course of symptoms. In this report, we describe a rare case of SAM in which a healthy man in his 40s with no underlying disease died after presenting with acute abdomen and intracranial hypertension, SAH, and hemorrhage of the greater omentum.

## 2. Case history

A Japanese man in his late 40s was found dead at home by his family. No resuscitation attempts were made. He initially presented to the emergency department late at night on the first day of illness with complaints of sudden-onset abdominal pain and headache.

During his visit, blood tests, abdominal ultrasound, and contrast-enhanced computed tomography (CT) scans of the thorax and abdomen were performed between the first and second days of illness. Laboratory findings revealed: white blood cell count, 14,400/µL; hemoglobin, 16.1 g/dL; hematocrit, 49.2%; platelets, 259,000/µL; AST, 21 IU/L; ALT, 19 IU/L; BUN, 16.5 mg/dL; and creatinine, 0.97 mg/dL. Arterial blood gas analysis shows a pH of 7.294, pCO_2_ of 58.2 mm Hg, pO_2_ of 26.5 mm Hg, HCO_3_^-^ of 27.6 mmol/L, and base excess of −0.4 mmol/L. No abnormalities were noted on CT imaging. Abdominal ultrasound revealed no evidence of cholecystitis or other identifiable causes of abdominal pain. His vital signs were within normal limits, and he was discharged home for outpatient management. On the third day of illness, he visited another hospital with a chief complaint of stomach pain. As the pain was mild, he was again managed at home. On the fourth day, the patient was found alive in the morning and was able to communicate with his family. However, he was found dead at home that night. He drank alcohol a few times a month and had no history of smoking. He had sought medical attention for stomach pain several times but had no medical or family history of sudden death. An autopsy was performed 5 days after his death. Written consent was obtained from the patient’s wife for the use of his personal and disease-related details in this case report.

## 3. Case findings

### 3.1. Autopsy findings

**Figure 1.**
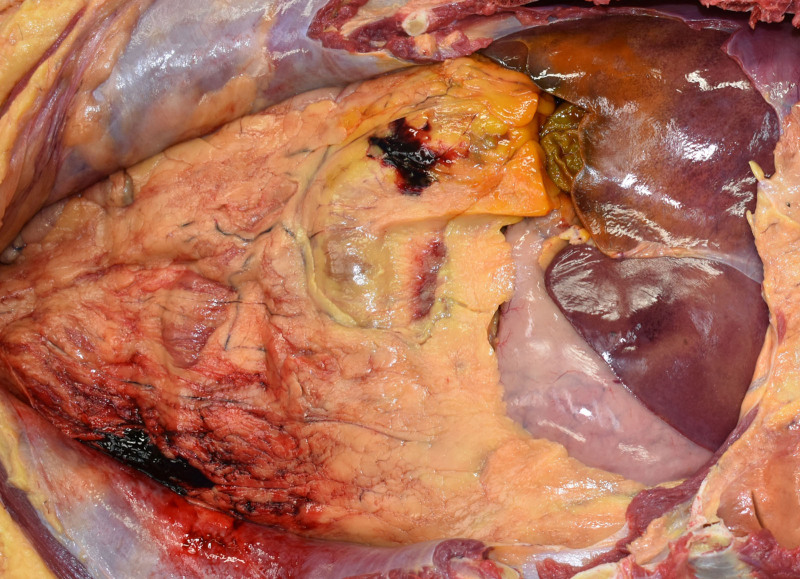
A photograph of the abdominal cavity. A few small hemorrhages in the greater omentum are noted.

**Figure 2.**
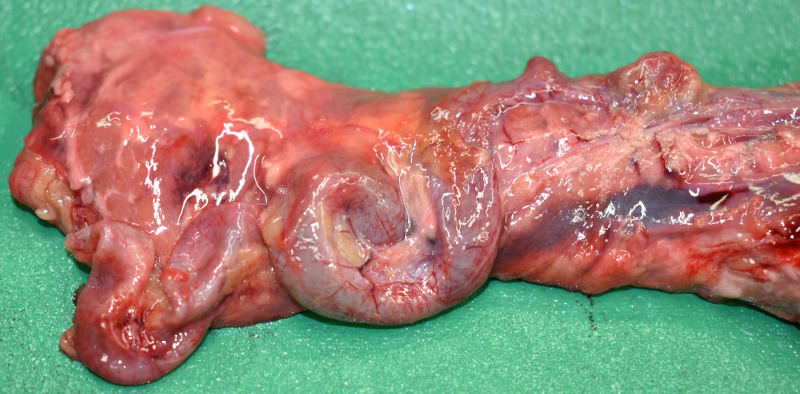
The deceased’s pancreas and the splenic artery. The splenic artery is highly tortuous and dilated, partly bulging like an aneurysm.

**Figure 3.**
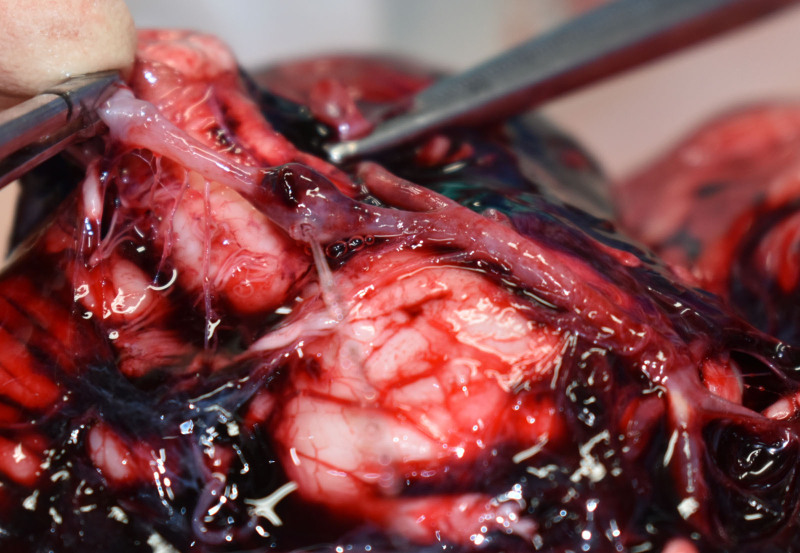
The base of the brain. A ruptured aneurysm, 7 mm in diameter, is evident in the left vertebral artery. A subarachnoid hemorrhage is also noted.

The patient’s height and weight were 174 cm and 68 kg, respectively; no body trauma was noted. A few small hemorrhages were observed in the greater omentum (Fig. 1). The spleen weighed 100 g, and the splenic artery was highly tortuous and dilated, partly bulging like an aneurysm (Fig. 2). The brain was enlarged to 1470 g, with a thick SAH observed from the base of the brain to the left and right Sylvian fissures. A ruptured aneurysm 7 mm in diameter was found in the left vertebral artery (Fig. 3). The anatomy of the cerebral arterial system showed no abnormalities, and atherosclerosis was mild. The heart weighed 380 g, and no scars or other abnormalities were found in the myocardium. Moderate luminal stenosis was observed in the coronary arteries. No other significant injuries or pathology were evident in the other organs.

### 3.2. Pathological findings

**Figure 4.**
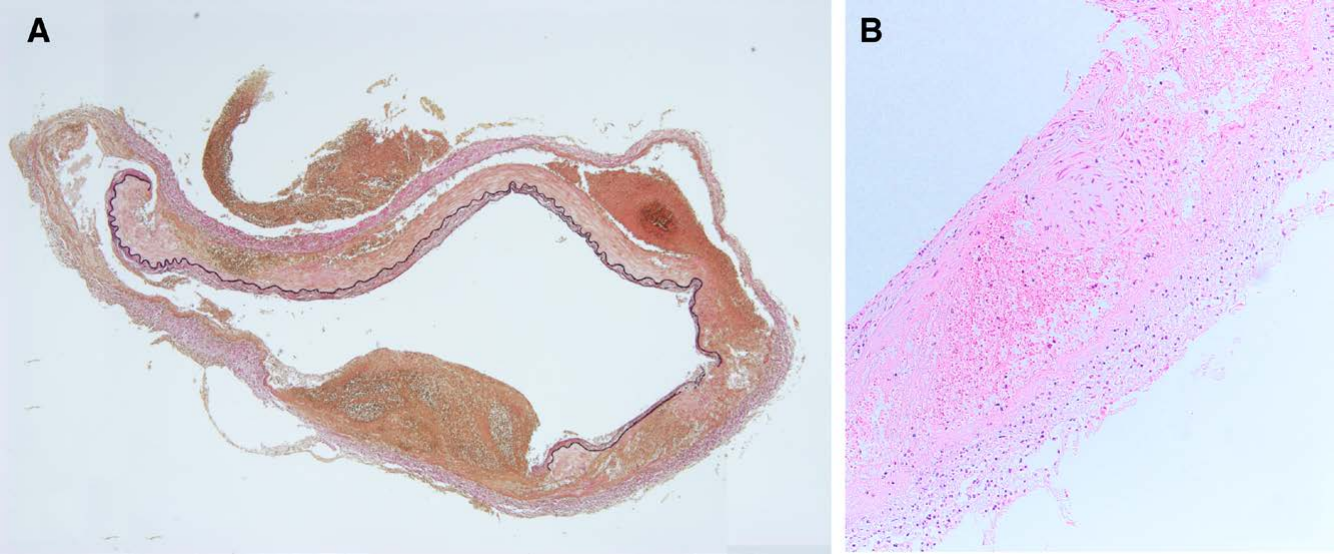
Histopathological photographs of the ruptured vertebral artery aneurysm. (A) The internal elastic membrane is disrupted at the extending portion of the vertebral artery aneurysm (Elastica van Gieson staining, low magnification). (B) Erythrocytes and inflammatory cells, including lymphocytes and neutrophils, are observed in the dissected lumen. The medial island remained in the tunica media of the dissected area, and vacuolar degeneration and mediolysis of smooth muscle cells are observed (hematoxylin and eosin staining, high magnification).

**Figure 5.**
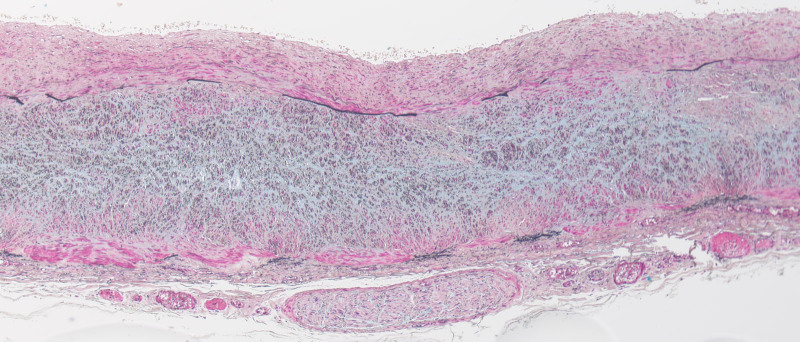
Histopathological staining of the splenic artery. The tunica media of the splenic artery is partially replaced by granulation tissue, with vacuolar degeneration of smooth muscle cells and disruption of the internal elastic membrane observed (Movat pentachrome staining, low magnification).

**Figure 6.**
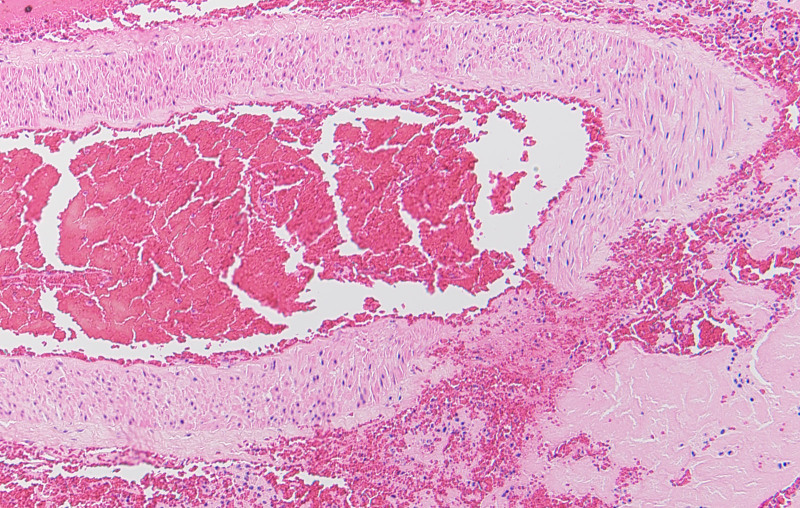
Histopathological staining of the ruptured artery in the great omentum. Transmural apoptosis of the tunica media smooth muscle cells is observed (hematoxylin and eosin staining, high magnification).

The internal elastic membrane was disrupted in an extended portion of the vertebral artery aneurysm (Fig. 4). Similar disruption was observed in arteries other than the aneurysms, noting erythrocytes and lymphocytes in the dissected lumen. The medial island remained in the tunica media of the dissected area. Vacuolar degeneration and mediolysis of smooth muscle cells were also observed (Fig. 4). The tunica media of the splenic artery was partially replaced by granulation tissue, with vacuolar degeneration of the smooth muscle cells and disruption of the internal elastic membrane (Fig. 5). Arterial rupture was observed in the hemorrhage area of the greater omentum, and transmural apoptosis of the smooth muscle cells in the tunica media was also found (Fig. 6). No inflammatory cell infiltrated the tunica media of any vessels, except near the dissected lumen.

### 3.3. Radiological findings

**Figure 7.**
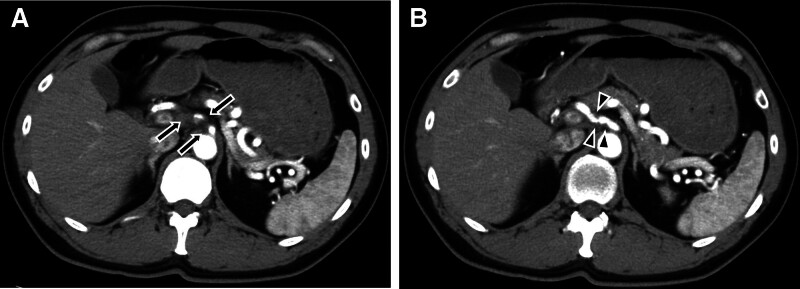
Two pictures of abdominal computed tomography angiography (A and B). The splenic artery shows high tortuousness. Slight wall thickening (arrows) and irregularly narrowed lumen (arrowheads) are noted at the celiac trunk, extending to the common hepatic artery and left gastric artery.

CT angiography (CTA) revealed slight wall thickening and an irregularly narrowed lumen at the celiac trunk, extending to the common hepatic and left gastric arteries (Fig. 7). No hematoma or ischemic changes were observed in the abdominal organs.

### 3.4. Diagnosis

The cause of death was determined as SAH resulting from a ruptured vertebral artery aneurysm associated with SAM.

## 4. Discussion

We present a unique case of a patient with multiple vascular lesions in the abdominal and cerebral arteries, with simultaneous hemorrhages, causing typical symptoms of SAM such as abdominal or flank pain, as well as other symptoms such as headache and vomiting. Although SAM does not always lead to death from intra-abdominal hemorrhage, SAH may coexist with it.^[5–9]^ In 2022, Tao et al^[7]^ reported 14 cases of intra-abdominal and SAH resulting from underlying SAM, including their own cases. SAM is a relatively rare disease, and simultaneous multiple hemorrhages, as in the cases reported by Tao et al, are even more uncommon. However, the condition is significant because of its serious consequences and potential for life-saving therapeutic intervention. Interestingly, the majority of those 14 cases were reported in Japan. However, since the cause of SAM is unknown, the reason for this apparent geographic clustering is also unclear. Notably, our report is also from Japan, suggesting that this trend may continue. To the best of our knowledge, no previous case of greater omentum hemorrhage as in the present case has been reported. The greater omentum hemorrhage seen in this case was difficult to detect because it was a minor finding.

Nevertheless, the reason behind the simultaneous occurrence of subarachnoid and great omentum hemorrhages in this case remains unclear. From the histological findings, it is uncertain whether the SAH was preceded by the great omentum hemorrhage or vice versa. Physiologically, blood pressure may increase from pain or other factors when one vessel fails, potentially causing an aneurysm at another site that is originally vulnerable to failure. If this occurs, diagnosing SAM becomes more challenging. The patient’s clinical course suggests that he developed a greater omentum hemorrhage first, followed by an SAH, which resulted in death. The rupture of aneurysms caused by SAM is fatal. However, the survival rate is as high as > 90% when treatment is provided at the appropriate time^[4]^; therefore, when a patient complains of unexplained headache or abdominal pain, it is crucial to consider SAM as a rare but potentially fatal disease in the differential diagnosis.

Although sex differences and the age of onset for SAM are not clearly established because of its rarity, it appears to be more common in males and typically affects middle-aged individuals.^[2,4]^ Abdominal pain is the most frequent symptom, occurring in approximately 80% of cases, followed by intra-abdominal bleeding in 50%.^[4]^ Thus, although abdominal pain is a very common symptom, SAM should be considered in the differential diagnosis when no other cause is identified – especially when age and sex align with known risk factors. Importantly, SAM is a systemic arterial disease that can present with a wide range of symptoms, including intracranial hemorrhage and cerebrovascular symptoms, which occur in approximately 12% of cases.^[4]^

Differentiation diagnoses for SAM include common causes of acute abdomen such as appendicitis, as well as similar arteriopathies (e.g., fibromuscular dysplasia [FMD], localized vasculitis of the gastrointestinal tract, isolated arterial dissection, and aneurysms), connective tissue disorders, and other vasculopathies.^[2,4,10]^ Clinically, imaging studies are essential for diagnosis when SAM is suspected. Although contrast-enhanced CTA is ideal, non-contrast CT may also be useful. Certain abdominal vessels – such as the superior mesenteric artery, splenic artery, and celiac artery – are commonly affected.^[2,4,10]^ Careful evaluation of these arteries on CT can aid in diagnosis. The most frequent imaging findings include aneurysms, arterial dissections, and rupture. In addition, multiple vascular lesions are found in 45% of cases, and cerebrovascular lesions are reported in 17%.^[4]^ Given that intracranial involvement is not uncommon, head imaging should be considered when SAM is diagnosed on abdominal imaging – especially in patients presenting with cerebrovascular symptoms such as headache.

FMD is particularly important to consider in the differential diagnosis.^[2,4,10]^ The characteristic “string of beads” appearance on imaging is typical of FMD but may also be observed in SAM. Biopsy plays a key role in the definitive diagnosis of SAM. The hallmark histopathologic feature is vacuolar degeneration of the arterial media, whereas FMD is characterized by fibrous or fibromuscular thickening of the arterial wall – features typically absent in SAM. However, biopsy is not always feasible. Therefore, distinguishing SAM from FMD requires understanding their clinical and demographic differences. FMD predominantly affects women and tends to have a relatively benign course, whereas SAM often presents acutely with serious complications such as arterial dissection or rupture. A comprehensive assessment that includes medical history is often necessary for diagnosis.^[2,4,10]^

Approximately half of patients with SAM present with abdominal bleeding at the time of diagnosis.^[4]^ In such cases, endovascular repair or surgery may be warranted. However, if no bleeding is detected and the risk of rupture is considered low, conservative management – such as blood pressure control – may be appropriate.^[2,4]^

In our case, the clinical course – abdominal pain, headache, and vomiting – was consistent with typical SAM. The patient’s age and sex were also characteristic. CTA revealed subtle arterial wall thickening and irregular luminal narrowing, findings suggestive of SAM. Histopathological examination confirmed arterial dissection and vacuolar degeneration of the arterial media. In vascular lesions without dissection or hemorrhage, no inflammatory cell infiltration was observed, consistent with SAM. Aside from the simultaneous intracranial and abdominal hemorrhages, the presentation was otherwise typical. Additionally, the splenic artery was markedly tortuous, which was disproportionate to the overall progression of systemic arteriosclerosis. Although arterial tortuosity is observed in conditions such as Loeys–Dietz syndrome, it is not specific and is thought by some to reflect age-related changes,^[4,11]^ whereas others argue against this interpretation, citing marked individual variation.^[12]^ Nevertheless, pronounced splenic artery tortuosity in a relatively young patient may support a diagnosis of SAM.

## 5. Conclusion

We encountered a case of SAM causing headache and abdominal pain simultaneously, resulting in death from SAH. SAM is under-recognized and may be difficult to diagnose, especially when the signs and symptoms are not typical. Although spatially separated pain is sometimes an indefinite complaint, understanding that SAM lesions occur in multiple vessels throughout the body is important because it can help prevent fatal bleeding. As in this case, SAM should be suspected when symptoms of intracranial hypertension – such as headache, nausea, and vomiting – are observed aside from abdominal or flank pain. We believe that identifying lesions in arteries throughout the body, including the head and neck region, and providing appropriate treatment can prevent mortality of patients with SAM.

## Acknowledgments

We would like to express our gratitude to Dr Yujiro Bise for providing valuable clinical data about the patient. We would like to thank Editage for the English language editing.

## Author contributions

**Data curation:** Miu Shimamoto, Kenji Ninomiya.

**Investigation:** Miu Shimamoto, Kenji Ninomiya, Akira Yogi.

**Resources:** Kenji Ninomiya.

**Writing – original draft:** Miu Shimamoto, Kenji Ninomiya.

**Writing – review & editing:** Miu Shimamoto, Kenji Ninomiya, Akira Yogi, Kazumichi Kakazu, Natsuki Ikematsu, Maki Fukasawa, Mio Takayama.
